# Ammonia Concentration in Ambient Air in a Peri-Urban Area Using a Laser Photoacoustic Spectroscopy Detector

**DOI:** 10.3390/ma15093182

**Published:** 2022-04-28

**Authors:** Mioara Petrus, Cristina Popa, Ana-Maria Bratu

**Affiliations:** Laser Department, National Institute for Laser, Plasma and Radiation Physics, 409 Atomistilor St., P.O. Box MG 36, 077125 Magurele, Romania; mioara.petrus@inflpr.ro

**Keywords:** atmosphere, air pollution, ammonia, laser photoacoustic spectroscopy

## Abstract

Measuring ammonia from the environmental air is a sensitive and prioritized issue due to its harmful effects on humans, ecosystems, and climate. Ammonia is an environmental pollutant that has an important role in forming secondary inorganic aerosols, the main component of fine particulate matter concentrations in the urban atmosphere. Through this study, we present a gas analyzer that utilizes the technique of laser photoacoustic spectroscopy to measure ammonia concentration in three different sites located in Magurele, (44°20′58″ N 26°01′47″ E, 93 m altitude), Romania, from March to August 2021 at the breathing level of 1.5 m above ground. The ammonia concentrations from the ambient air were elevated in summer (mean of 46.03 ± 8.05 ppb (parts per billion)) compared to those measured in spring (18.62 ± 2.92 ppb), which means that atmospheric temperature affects ammonia concentrations. The highest mean ammonia concentrations occurred in August, with an ammonia concentration level of 100.68 ± 11.12 ppb, and the low mean ammonia concentrations occurred in March, with an ammonia level concentration of 0.161 ± 0.03 ppb. The results confirm that meteorological characteristics (i.e., temperature) and motor vehicles are major contributors to the elevated ammonia levels during the monitoring period.

## 1. Introduction

The composition of the air in the atmosphere is constantly changing. Some of the substances present in the atmospheric air react with others forming “secondary” pollutants harmful to our health and the environment [1]. Particulate matter (PM) are fine particles that can have a primary origin when they are directly emitted into the atmosphere from road traffic and car exhaust gases and a secondary origin when they are precursors of pollutants (e.g., NH_3_, NOx, and SO_2_) after photochemical reactions in the atmosphere [1,2,3,4,5]. Some particles with less than 10 µm (PM_10_) in diameter can go deep into your lungs, and some may even go into your bloodstream, and from these, those particulate matter with less than 2.5 µm in diameter (PM_2.5_) pose the greatest risk to health [6]. In the atmosphere, ammonia can react with acidic species, such as sulfuric acid (H_2_SO_4_), nitric acid (HNO_3_), and hydrochloric acid (HCl), and produce PM_2.5_ such as ammonium sulfate ((NH_4_)_2_SO_4_), ammonium nitrate (NH_4_NO_3_), and ammonium chloride (NH_4_Cl) [7].

Ammonia is an irritating gas to the skin, respiratory tract, digestive tract, or eyes and plays an important role in acidification and eutrophication with impacts that produce a decrease in biodiversity and changes in species composition [8,9]. Agriculture has a major contribution to the presence of environmental ammonia, about 80%, from the use of nitrogen-based fertilizers and fertilization with urea [3,4], but wastewater treatment, wild animals, human excreta, traffic, catalytic converters, and biomass burning represent other sources [2,5,6,7,10].

In recent decades, in urban environments, the application of urea as a selective catalyst for reducing NOx emissions has increased due to the toxic effects of environmental ammonia on humans and ecosystems, it is necessary to measure environmental ammonia, but this is also a challenge due to the low concentrations of ammonia in the atmosphere and the relatively high concentrations of potentially interfering atmospheric components, especially water vapor [6,7].

New methods for ammonia concentration detection from the atmospheric air have been developed: chemical ionization mass spectrometry (CIMS) [11,12], quantum cascade tunable infrared laser differential absorption spectrometer (QC-TILDAS) [13,14], differential optical absorption spectroscopy (DOAS) system [15,16,17,18], photoacoustic (PA) technique [19,20,21,22], or gas sensors based on graphene-based material [23,24]. Remote sensing has been preferred in the last years due to the extensive geographic coverage and capability of capturing spatiotemporal variations in columns and surface NH_3_ concentrations of satellites. Important attention was given to Tropospheric Emission Spectrometer (TES), but due to the limitations of the TES related to its small geographic coverage and consequent inability to provide daily coverage, was preferred the monitoring by the Infrared Atmospheric Sounding Interferometer (IASI) [25,26]. The ammonia monitoring using satellite remote sensing has limitations such as discontinuous temporal measurements caused by the fact that these instruments are not onboard geostationary satellites, the presence of clouds affecting the measurements, the nighttime measurements being reduced, but are some problems related to the inference of ammonia from the radiances detected [27,28,29]. Important attributes for trace gas analysis such as high sensitivity and selectivity, large dynamic range, high accuracy and precision, good temporal resolution, and versatility are provided by laser photoacoustic spectroscopy (LPAS). LPAS has the advantage of tracing gases locally and in laboratory studies, can also achieve a high sensitivity within a small volume of gas, and is a background-free technique [30]. M.B. Pushkarsky et al. developed an ambient sensor that uses resonant photoacoustic spectroscopy and a line-tunable CO_2_ laser for ammonia detection with a sensitivity of ppt (parts-per-trillion) level [22]. In environmental pollutants, monitoring is desirable for a detector with a multi-component capability. This opportunity has been realized by using near-IR lasers due to their characteristics such as low cost, continuous tunability, and detection of a large number of interest molecules (CO_2_, CO, H_2_O, NH_3_, C_2_H_4_, C_6_H_6_, O_3_, etc.). In this way, the laser photoacoustic spectroscopy sensors become promising tools in multi-component trace gas detection from ambient air [31,32,33,34]. In photoacoustic spectroscopy, mixture samples are composed of gases that can interfere with the target molecules, and in order to eliminate this issue, several more studies have been carried out [35,36]. One of the multiple ambient gases that can be detected by LPAS with high sensitivity is ammonia [32].

Ammonia is an important air pollutant with negative effects on both human health and the environment and reacts with other substances to form PM_2.5_ (e.g., ammonium sulfate and ammonium nitrate), which represent up to 80% of PM_2.5_ particles [37,38], and PM_2.5_ concentration in Magurele air is currently between 2.5 and 3.2 times above the World Health Organization (WHO) annual air quality guideline value. Despite the danger it poses, in Romania, there is a lack of data on the concentration of ammonia in the atmospheric air, being poorly monitored and understood, with only a few studies and measurements on the concentration of ammonia in the atmosphere [39,40,41]. Through this study, we quantified the concentration of atmospheric ammonia and temporal variations at 1.5 m above the ground, in three different locations in environmental structures, from March to August 2021, using a laser photoacoustic detector. Characteristics of temporal and spatial ammonia concentrations distributions among the three different locations from the monitoring period are compared and discussed, together with information about weather conditions.

## 2. Materials and Methods

### 2.1. Human Settlement Layer

Romania is situated in the south-eastern part of Central Europe inside and outside of the Carpathians Arch, on the Danube lower course. Romania’s climate is a transitional temperate-continental one with oceanic influences from the West, Mediterranean modulations from the South-West, and excessive continental effects from the North-East. The climate in Romania suffered changes in the last years, most of those related to the mean annual increase in temperature in the Eastern and Southern regions of the country (e.g., hotter summers with more frequent heatwaves) and a reduction in mean precipitation in the Southern part of Romania [42,43,44].

**Figure 1:**
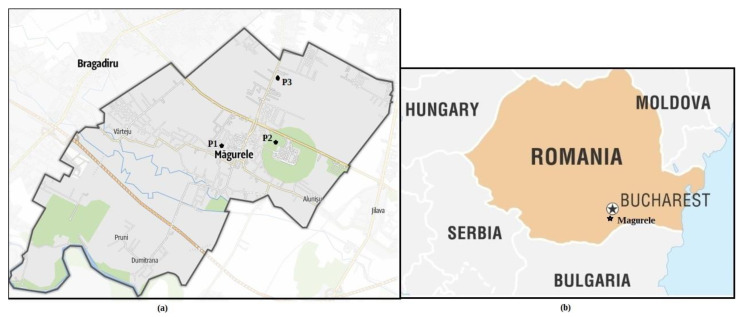
The geographical location of sampling sites: (**a**) Monitoring points P1, P2, and P3; (**b**) the location of Magurele on the map of Romania.

Monitoring of ammonia in atmospheric air was performed in Magurele city (44°20′58″ N 26°01′47″ E, 93 m altitude), Romania (see Figure 1).

The determination of the ammonia concentration in the ambient air was performed in the spring and summer seasons between March and August 2021. The gas samples were collected on the workdays from Monday to Friday, at a specific time, in the interval 08:30–11:30 AM and in the evening in the interval 07:30–08:30 PM at three different points. The three monitoring locations are P1—44°21′02.7″ N 26°01′42.0″ E; P2—44°21′10.4″ N 26°02′31.0″ E; and P3—44°22′09.6″ N 26°02′34.2″ E.

Magurele is a city located in Ilfov County, located in the southwestern vicinity of Bucharest, at 10 km from the capital, being a satellite city of the Capital. This city is in continuous development; new residential neighborhoods have been developed in the last few years, the population is young, and the number of children increases every year. For this research were chosen the three locations were because of their difference in environmental structures. Over 300 economic agents have their headquarters and work points in the city of Magurele, and there are also new national research institutes in the field of physics and the Physics Faculty. The P1 point is located inside the city, at a roundabout and 150 m from a school where over 1000 students study, and also 150 m from a kindergarten with over 300 children with ages from 3 years to 6 years. The P2 point is located in a small forest (oak *Quercus robur* and black locust *Robinia pseudoacacia* are the predominant trees) near the national institute for nuclear physics and is surrounded by two heavily trafficked roads, one of these being the Bucharest ring road. The P3 point on the road that bounds Magurele from Bucharest is located in an industrial area, gas stations, and a concrete station, where the greenery areas are missing.

### 2.2. Air Samples and Method

**Figure 2:**
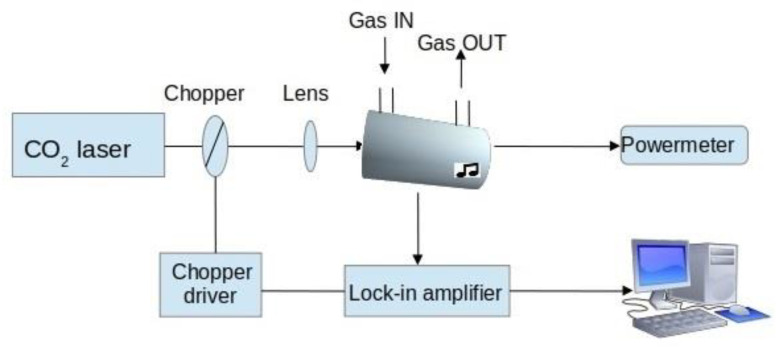
Schematic representation of the CO_2_LPAS detector used for environmental ammonia measurements.

A CO_2_ laser photoacoustic spectroscopy (CO_2_LPAS) system home-made with a ppb detection limit was used for measurements of ambient ammonia. The set-up of the instrument is shown in Figure 2 and described in Dumitras et al., 2007 [45]. Air samples were taken using a specialized container/bag, at the breathing level of 1.5 m above ground level, between March and August 2021, during the workdays from Monday to Friday, and analyzed in the laboratory using the LPAS system.

The LPAS system, as can be seen in Figure 2, comprises a CO_2_ laser radiation source, a photoacoustic (PA) cell where the gas sample is enclosed and analyzed, a vacuum/gas handling system, and a detection unit.

The CO_2_ radiation source is a home-built laser, line-tunable and frequency-stabilized, that emits continuous-wave radiation with an output power in the range of 2–5 W, tunable between 9.2 and 10.8 μm on 57 different vibrational-rotational lines. The requirement for gases molecules to be detected is that molecules have to possess a high absorption strength and a characteristic absorption pattern in the wavelength range of the CO_2_ laser. The PA cell is made of stainless steel and Teflon to reduce the out-gassing problems and consists of an acoustic resonator (pipe), windows, gas inlets, and outlets. Inside the PA, traces of gas can absorb the laser radiation, and the absorbed energy is released into heat, which creates an increase in pressure inside a closed volume. By modulating the laser beam with a mechanical chopper model DigiRad C-980 whose operating frequency is the same as that of the PA cell (564 Hz), pressure waves are generated and detected with four sensitive miniature microphones (Knowles electrets EK-303 or EK-23024, sensitivity 20 mV/Pa) mounted in the cell wall. Their electric signal is fed into a dual-phase, digital lock-in amplifier, and its filtered output signal is introduced in the data acquisition interface. All experimental data are processed in real-time and stored by a computer. A software program for graphics and instrumentation permits obtaining and processing of the experimental results via a National Instruments acquisition card (NI cDAQ-9174) under computer control. The absolute trace gas concentrations are processed by the computer, and the results are displayed on the screen.

Before entering the PA cell, the CO_2_-cw laser beam is modulated by a mechanical chopper (DigiRad C-980 and C-995 (30 aperture blade), Terahertz Technologies Inc., Oriskany, NY, USA) that operates at the resonant frequency of the PA cell (564 Hz) and is focused by a ZnSe lens (*f* = 400 mm), and introduced in the PA cell where is locally absorbed by IR active molecules. After passing through the PA cell, the laser beam power is measured by a radiometer (Rk-5700 from Laser Probe Inc. (Utica, NY, USA) with a measuring head RkT-30) connected to a data acquisition interface module together with a lock-in amplifier ((Stanford Research Systems model SR 830), Sunnyvale, CA, USA) which filters and amplifies the signal from microphones. The lock-in amplifier also gives the amplitude and phase of the chopper phase-synchronized PA signal. The acquisition interface is connected to a computer where all experimental data are processed in real-time and stored. The software allows the display of several parameters, such as the values for the PA voltage, average laser power after chopper, and the trace gas concentration. The number of the absorbing molecules (or the trace gas concentration) from the PA cell is proportional to the amplitude of the PA signal, and the measured trace gas concentration is given by:

*c* = *V*/(*αP_L_CS_M_*) (1)

where: *c* (atm) is the trace gas concentration (1 ppbV (parts-per-billion by volume) = 10^−9^ atm; 1 pptV (parts-per-trillion by volume) = 10^−12^ atm), *V* (V) is the PA signal (peak-to-peak value), α (cm^−1^ atm^−1^) is the gas absorption coefficient at a given wavelength, *C* [Pa cmW^−1^] is the cell constant, *P_L_* [W] is the cw laser power before chopper, and *S_M_* [V Pa^−1^] is the microphone responsivity.

The vacuum/gas handling system is a very important part of PA measurements due to its role in ensuring the purity of the PA cell or in the evacuation of the entire gas mixture from the system, including the PA cell, either completely or in different sections, or to monitor the total gas pressure. This PA component, thanks to the Teflon/stainless steel system, can perform several functions without the need for disconnections. This system is built with Swagelok flexible PFA Teflon tubing (PFA-T6M-1M-30M, 6 mm), toggle valves (SS-1GS6), and union tees (SS-6M0-3), ensuring the sample purity along with proper isolation of the gas sample inside the PA cell, and a minimum mechanical vibration at PA cell. The gas or gas mixture is introduced into the system at a controlled flow rate of 600 sccm (standard cubic centimeters per minute). A series of manometers allows the gas pressure to be read inside the PA cell, as well as in different segments of the system.

**Figure 3:**
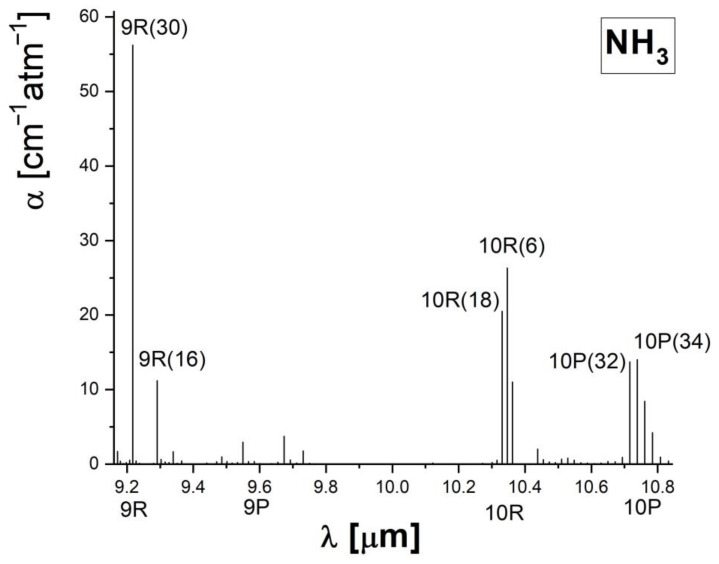
Ammonia absorption coefficients of CO_2_ laser wavelengths lights on 9P, 9R, 10P, and 10R branches [46].

The number of detectable molecules is related to the spectral overlapping of the CO_2_ laser emission with the absorption bands of the trace gas molecules. The ammonia concentrations from the atmospheric air were determined on the 9R(30) line of the CO_2_ at the wavelength λ = 9.22 μm, where the ammonia present the highest absorption coefficient α(NH_3_) = 57.12 cm^−1^ atm^−1^ (see Figure 3) [46]. Figure 3 shows the other ammonia absorption coefficients, covering the branches P and R, and it can be observed that ammonia presents weaker absorption coefficients at other CO_2_ laser lines [46].

The CO_2_LPAS system performed a multi-component analysis and, in this situation, the interference of other substances may affect the theoretical limit of detection. In the CO_2_ laser spectral range, a large number of absorbent interferences from the atmosphere, such as water vapors and carbon dioxide. To increase the accuracy of the LPAS method for measurements of ammonia, we used a potassium hydroxide (KOH) trap of 120 cm^3^ (filled with a KOH-based scrubber) inserted between the sampling cell and the PA cell for reducing the carbon dioxide and water vapors concentration [47]. Because ammonia is a highly adsorbing compound and the results of successive measurements are often altered by the molecules previously adsorbed on the pathway and cell wall, an intensive cycle of nitrogen 6.0 (purity 99.9999%) washing was performed between samples to have a maximum increase of 10% for the background PA signal (to ensure the quality of each measurement). Nitrogen gas is transparent to the CO_2_ laser radiation, and a good cleaning means a PA signal of around 20 µV/W.

Meteorological data (air temperature, relative humidity, atmospheric pressure, wind speed, and direction) were obtained from a meteorological station model Eurochron WS1080 used to measure the weather parameter in the three locations. The data measured by the external sensors are transmitted wirelessly to the central unit. The weather station is equipped with an internal barometric sensor and calculates a forecast for the next 12–24 h based on records of changes in atmospheric pressure. All-weather data recorded by the base station and external sensors can be saved on a PC at intervals set by the user.

## 3. Results

**Figure 4:**
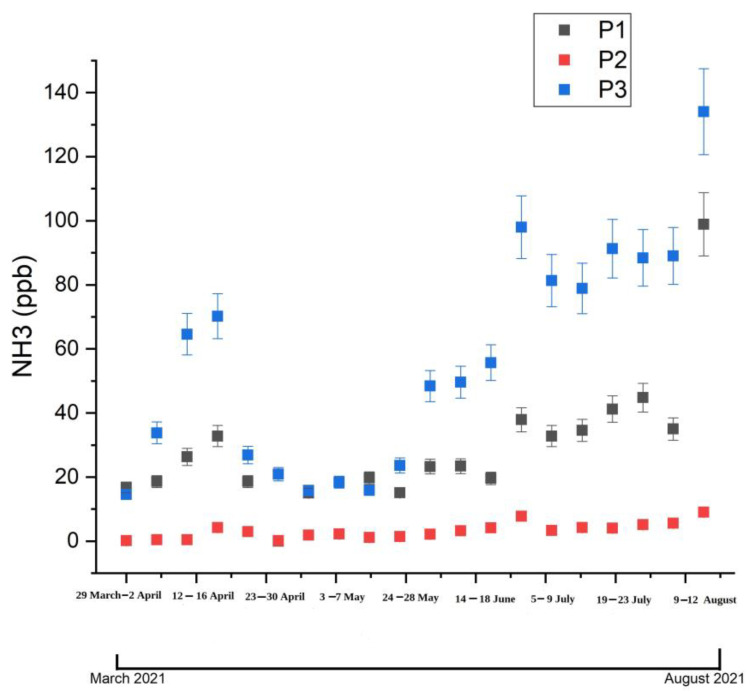
Average weekly ammonia concentration in the three-monitoring points P1, P2, and P3 throughout the period March–August 2021.

As mentioned above, measurements of ammonia concentrations from three different locations, P1, P2, and P3, used a CO_2_LPAS detector. The purpose of this study was to find the pattern for the ammonia concentration during the spring and summer seasons and to evaluate the influence of meteorological parameters on ammonia concentrations level. During the monitoring period, the highest ammonia concentrations occurred in August in the P3 site with 100.68 ± 11.12 ppb, and the lowest ammonia concentrations occurred in March in the P2 site with 0.161 ± 0.03 ppb. In the spring season, the average ammonia concentration was 20.59 ± 1.48 ppb in the P1 site, 1.71 ± 0.38 ppb in the P2 site, and 33.56 ± 5.71 ppb in the P3 site. In the summer seasons, the average ammonia concentration was 43.10 ± 8.39 ppb in P1, 5.41 ± 0.70 ppb in P2 site, and 89.58 ± 7.77 ppb in P3 site. In the P1 point, the minimum ammonia concentration measured was 15 ppb, and the maximum ammonia concentration was 98.9 ppb. The minimum ammonia concentrations in the P2 point were 0.161 ppb, and the maximum was 9.02 ppb. In the measuring point P3, the minimum ammonia concentration was 14.6 ppb, and the maximum ammonia concentration was 134 ppb. These measurements were used to estimate the behavior of ammonia levels during the weekdays in order to make a pattern of ammonia concentration behavior according to meteorological parameters. Figure 4 shows the average weekly concentration of atmospheric ammonia in the three monitoring locations, P1, P2, and P3, during the monitoring period in the spring and summer seasons. This figure shows a maximum average concentration determined in the P3 area and a minimum in the P2 monitoring area. P3 location is situated in an area where over 300 economic agents are based in this area, which means a large number of people who work and transit the area by car or public transport, and the P2 location is situated in a small and even if it is surrounded by two heavily trafficked roads, one of which is the ring road of Bucharest has the lowest ammonia concentration, and this means that the high contribution to these lowest values is represented by the presence of the trees.

**Figure 5:**
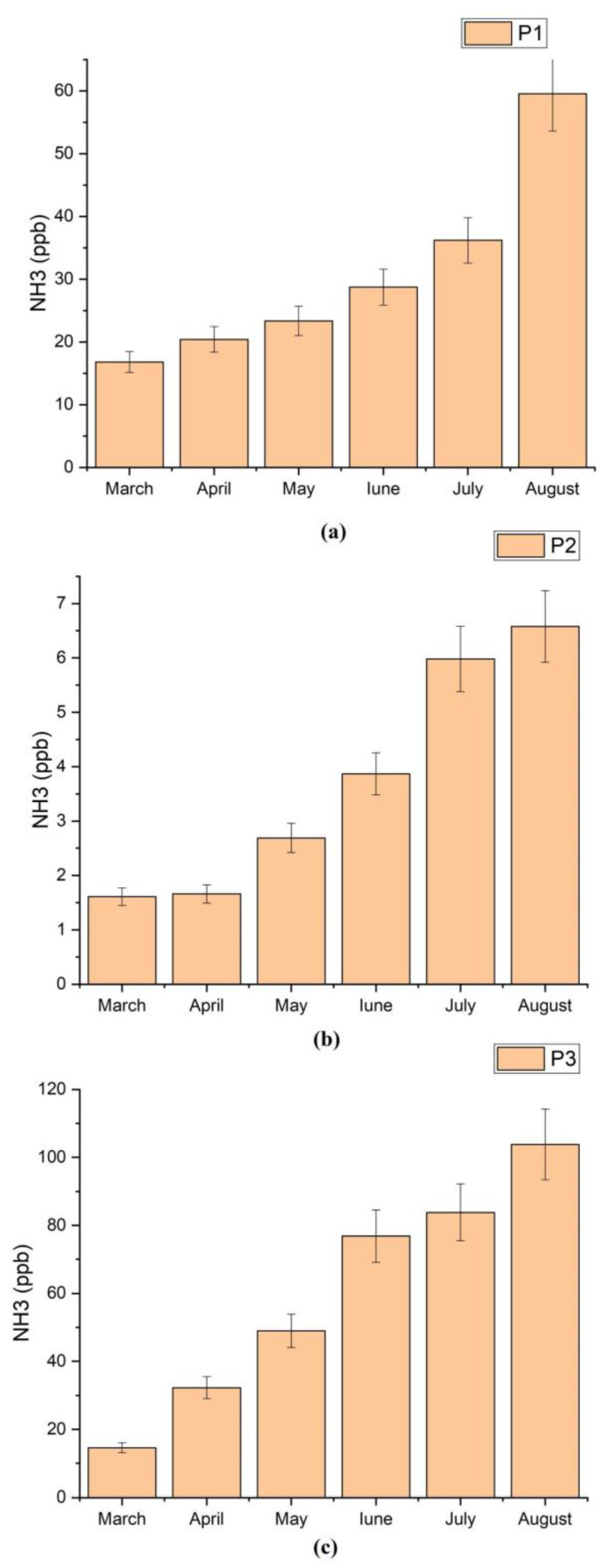
Monthly average ammonia concentration during the period March–August 2021: (**a**) P1 monitoring point, (**b**) P2 monitoring point, (**c**) P3 monitoring point.

Also, in order to predict the ammonia pattern during the two seasons, spring and summer were realized the average monthly ammonia concentration over the entire monitoring period (see Figure 5). From this figure can be observed that the highest ammonia concentrations were measured in the summer period. Thus, an increase in ammonia concentration can be observed during the summer in all three measurement points. The ammonia concentrations from the ambient air were elevated in summer (mean of 46.03 ± 8.05 ppb) compared to those measured in spring (18.62 ± 2.92 ppb), and the ammonia level increased can be correlated with ambient temperature increase.

**Figure 6:**
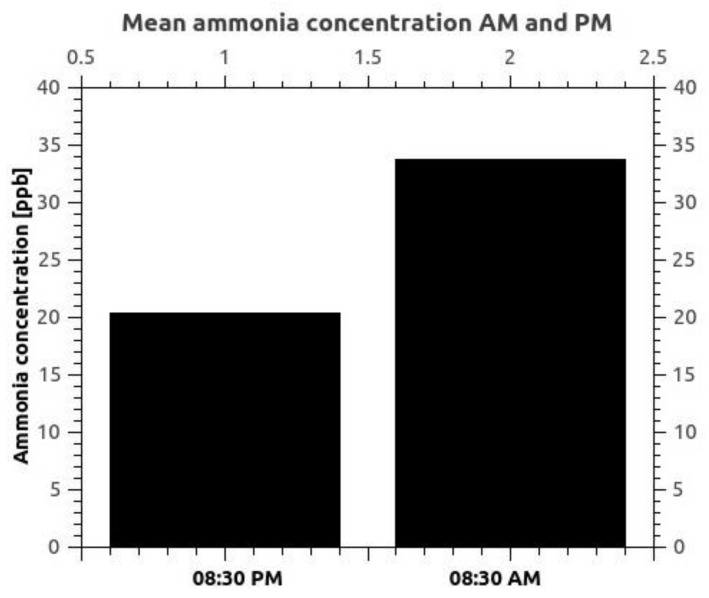
Diurnal variation of the average ammonia concentrations in the interval 08:30–11:30 AM and 07:30–08:30 PM in the three points P1, P2, and P3 over the entire monitoring period March–August 2021.

Figure 6 shows the plot that indicates the diurnal profiles of ammonia concentration at 08:30 AM (EEST) and 08:30 PM (EEST) and observed higher ammonia values in morning hours correlated with the rush hours as a consequence of vehicles emissions. Furthermore, many studies have observed that with the introduction of three-way catalytic converters in vehicles to reduce the toxic gases into less-toxic pollutants, the ammonia concentration in the atmosphere of the urban area increased significantly [8,9,10]. Monitoring points P1, P2, and P3 are located in areas where there is no agricultural activity nearby, and as can be seen in Figure 6, there is a relationship between the atmospheric ammonia concentration and the vehicle rush hours.

**Figure 7:**
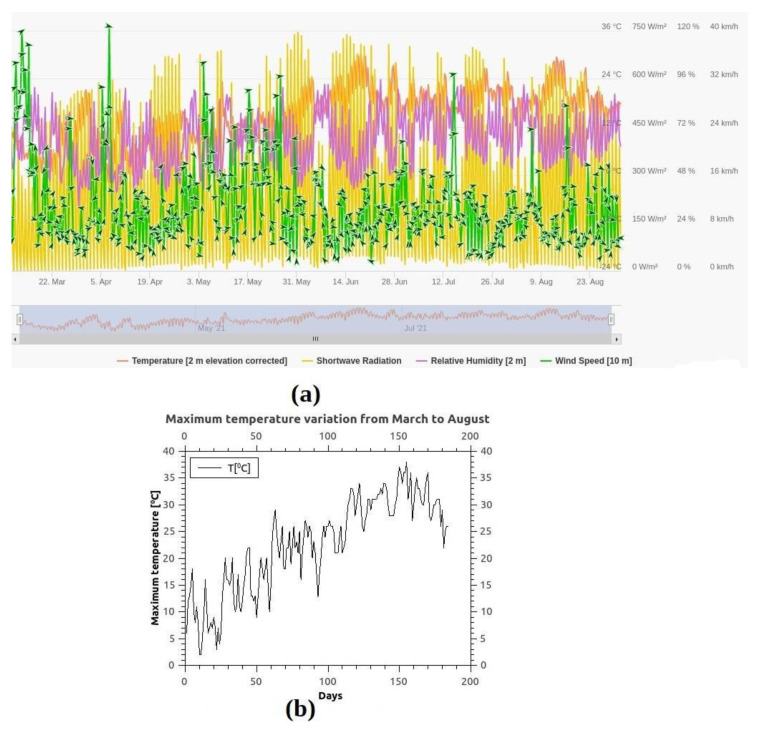
Meteorological parameters variation in Magurele, Romania from March to August 2021: (**a**) Temperature, shortwave radiation, relative humidity, and wind speed (meteoblue.com, accessed on 17 November 2021), (**b**) Variation of the maximum daily temperature in Celsius degree.

**Figure 8:**
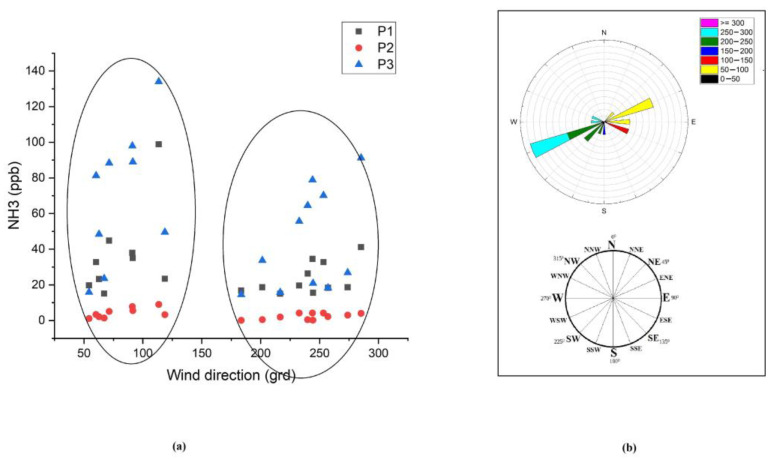
Dependence of ammonia on wind direction: (**a**) Ammonia concentration distribution in P1, P2, and P3 points as a function of wind direction; (**b**) The wind rose from March-August.

The atmospheric parameters were analyzed to see if there is an influence on ammonia concentration. Figure 7 shows the variation of some meteorological parameters such as temperature, humidity, shortwave radiation, and wind speed from March to August 2021 (data provided by meteoblue.com), and also the maximum temperature variation during the measuring period. The wind speed and direction influence on ammonia concentration were analyzed. Figure 8 presents the ammonia concentrations distribution in the P1, P2, and P3 measuring points as a function of the wind direction, and it can be observed that the NH_3_ concentrations are correlated with the wind direction from the E, ENE (54–92 degrees), and from the S, WSW direction (180–274 degrees), which means that the wind direction represents an important parameter in the ammonia distribution in Magurele.

**Figure 9:**
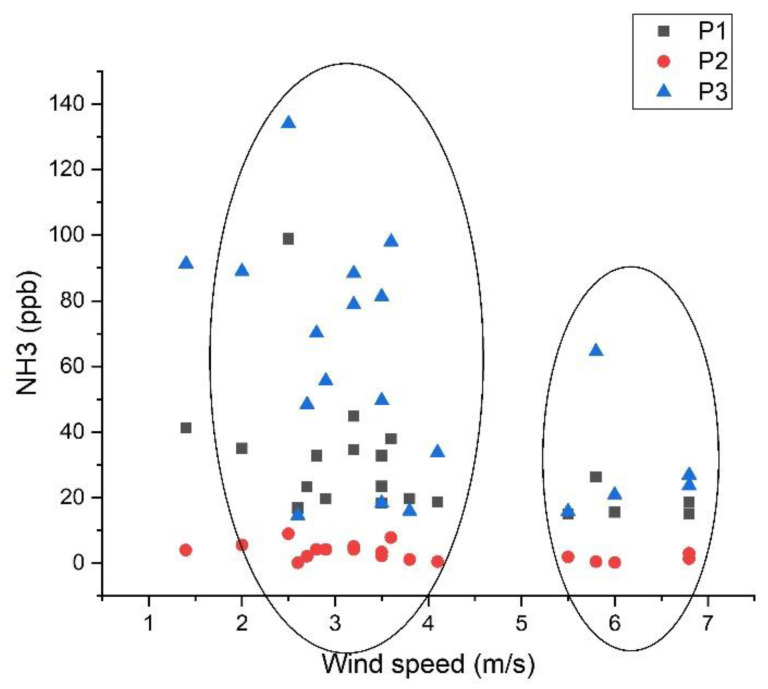
Ammonia concentrations are distributed in P1, P2, and P3 locations as a function of wind speed for the spring and summer seasons.

The distribution of the daily averages of ammonia concentrations in the P1, P2, and P3 measuring points for the entire period according to wind speed can be seen in Figure 9. In this figure, it can be observed that part of the ammonia concentrations is distributed in the range of 2.5–4 m/s, but high values of ammonia concentration can be observed at the measuring point P3, where higher wind values are presented between 6 m/s and 7 m/s.

Around the world, ground-, air-, and space-based sensors and ground techniques focus on ammonia monitoring, sources, and molecules movements. Table 1 compares ground level-atmospheric ammonia concentrations obtained with different sensors, and as can be observed, the ammonia concentration from the ambient air in Magurele is elevated compared to other studies.

## 4. Discussion

The analysis made by WHO’s International Agency for Research on Cancer (IARC) comes to the conclusion that ambient air pollution is carcinogenic to humans, the particulate matter being mostly associated with the increased number of humans with cancer, especially those with lung cancer [58]. According to the data shown by the EEA for the 2006–2016 period, 95–99% of the EU urban population is exposed to high polluted gases concentration that exceeds the limits imposed by WHO [59,60].

In our study, the highest values of the ammonia concentration in the atmosphere were determined in the P3 location during the summer season, with a mean value of 54 ± 34 ppb. Point P3 is located in an open area, without areas with plants or areas with trees, and in this monitoring point, over 300 economic agents are based in this area, which means a large number of people who work and transit the area by car or public transport. The point P2 is situated in a small forest (composed of trees species such as *Quercus robur* and *Robinia pseudoacacia*), and even if it is surrounded by two heavily trafficked roads, one of which is the ring road of Bucharest/the country’s capital here, where we recorded the lower ammonia concentrations, with the mean concentration of 3.19 ± 2.43 ppb. Compared to other studies, the ammonia concentrations detected using our LPAS system in the spring and summers seasons are higher.

According to Grigorieva and Lukyanets, higher concentrations of PM_2.5_ and PM_10_, O_3_, CO, and NO_2_ are a consequence of higher temperatures and light intensities on hot days and are associated with an increase in patients with chronic obstructive pulmonary disease, daily hospital emergency transports for asthma, acute and chronic bronchitis, and premature mortality caused by respiratory disease [61]. Some studies support urban forests and the construction of green infrastructure as a measure to reduce pollution while at the same time improving air quality and life [62,63].

Through this study, the atmospheric parameters’ influence on ammonia concentration was studied. Some studies present a high ammonia level on hot days, and these levels are affected by meteorological factors, i.e., air temperature, relative humidity, wind speed, and direction [64,65]. According to the seasonal division standard in Romania, March to May is spring, and June to August is summer. In Magurele, the spring of 2021 was with low temperatures and very large amounts of precipitation, and the summer of a very hot one, with tropical nights and precipitation in very small quantities, even absent in July and August. The temperature interval in spring was from 20 °C to 30 °C during the day and from −5 °C to 14 °C during the night, with the relative humidity (RH) was in March from 77.8% to 93.0%, in April from 59.0% to 96.2%, and in May from 50.4% to 94.0%. In consequence, lower temperature and higher humidity conditions are favorable for the conversion of ammonia to particle ammonium [48].

In 2021, the summer season was characterized by hot days, with temperatures between 21 °C and 38 °C, a very hot night in July and August, and the rainfall regime was very deficient. According to our measurements, the ammonia concentrations were high in the summer period compared to the spring period, and this can be due to an increase in the ambient temperature. It was also analyzed whether there is an influence of wind speed and direction on the ammonia concentration. From the climatic conditions, apart from temperature, wind speed and direction are meteorological parameters that influence the ammonia concentration [66]. The diurnal trends of ammonia were dependent on the air temperature and were affected by wind direction, indicating the influence of local and regional sources [64,67].

In Bucharest are located several power plants, mainly fossil fuel-based, these being important sources of particles air pollution [68]. The total annual fuel consumption per power plant, according to the Romanian Ministry of Environment, is 93.59% natural gas, 6.4% fuel oil, and 0.01% diesel fuel [69]. The environmental air in Magurele may also be influenced by cement stations which operate at a high rate in the warm seasons, one of these being located 100 m from the P3 measuring point.

The ambient ammonia concentrations in the atmosphere were correlated with different sources, and in residential areas, the sources are non-agricultural sources such as heating with natural gas, motor vehicles, and natural gas combustion for electricity generation [70,71]. The temporal patterns from these results showed that motor vehicles are the largest source. According to Sun et al., in cities, the ammonia sources are different, and this atmospheric ammonia concentration is caused by the intense presence of cars [72]. With the introduction of catalytic converters in car engines, ammonia concentrations in urban or high-traffic areas have increased, and this phenomenon was observed by many studies [73,74]. According to our study, the important source of atmospheric ammonia is represented by motors vehicles. The P1 and P3 sites are situated in an area with heavy car traffic; moreover, the P2 site is near two roads heavily used by cars, one of them being the ring road of Bucharest, but is but it is surrounded by trees is located in a small forest, but present low ammonia levels.

The research conducted by S. Banzhaf et al. shows that environmental ammonia molecules contribute to both formation of PM_2.5_ and the deposition of reactive nitrogen [75]. In the reports published by IQAIR with the most polluted PM_2.5_ air in 2020, Romania ranks 57th among 106 countries, with an average concentration of 15.8 µg/m^3^. According to IQAIR, the PM_2.5_ concentration in Romania’s air is currently 1.6 times above the World Health Organization (WHO) annual air quality guideline value, and in Magurele city, the PM_2.5_ concentration in air is currently 4.3 times above the WHO annual air quality guideline value. Ammonia concentration in PM_2.5_ formation is around 8% to 11%, according to other studies, while SO_2_ has a contribution between 9% to 11%, and NOx from 5% to 11% [48,76]. NH_3_, SO_2_, and NO_2_ are considered the primary precursors for the formation of PM 2.5 ammonium sulfate [77].

According to Romanian legislation, the level of PM_2.5_ suspended particles must not exceed the annual value of 20 ug/m^3^ (28.57 ppb) per year. The presence of a higher concentration of PM_2.5_ in the atmosphere of Magurele can be correlated with the elevated ammonia concentration obtained with our CO_2_LPAS detection system. The correlation between environmental ammonia and the presence of PM_2.5_ in the urban ambient air was observed in other studies [7,78]. By knowing the characteristics of the meteorological parameters, especially temperature and the wind direction and speed, the authorities can adopt measures to prevent high ammonia concentrations.

## 5. Conclusions

The ammonia molecule is a sticky one, and the presence of ammonia in the ambient air represents a challenge for monitoring detection systems, and urban ammonia emissions are underestimated. Analysis of ammonia concentrations from the environmental air in Magurele, Romania, in three locations was realized using a sensitive CO_2_LPAS detector. The CO_2_ laser wavelength has been carefully selected on the 9R(30) laser line at λ = 9.22 μm, where the ammonia present the highest absorption coefficient α(NH_3_) = 57.12 cm^−1^ atm^−1^, to achieve strong ammonia absorption while maintaining the potential for interference from other species as low as possible.

The three sites showed higher NH_3_ levels in summer than in spring, the highest values of ammonia concentrations were measured in the summer days of 2021 at point P3, which is located in an industrialized and heavily circulated area, and the ambient temperature is one of the factors that influence the environmental ammonia concentrations. Another important factor in the reduction of ground-level ammonia concentration is represented by the tree’s presence; the lowest ammonia values were determined in P2, a location that is surrounded by threes. There were stronger temporal patterns of ambient air ammonia, and according to our plots, the ammonia concentration is elevated in the morning compared to evening measurements, thus being associated with the rush hours and with the emissions caused by motor vehicles.

Trees play a decisive role in the presence of ground-level ammonia, and from this study, it can be concluded that the presence of an area with trees greatly reduces the ground-level ammonia concentration than in industrialized areas.

Compared to other studies, the ammonia concentrations in Magurele were found higher, and these high concentrations present in the surrounding air can be correlated with the presence of the high concentration of PM_2.5_.

Ammonia has become an important compound in ambient air pollution, and new techniques are needed to monitor this species. Compared to other trace gas detection systems, the CO_2_LPAS technique has the advantage of tracing gases locally with high sensitivity, selectivity, and multi-component capability, being able to different environmental pollutants.

Further studies are necessary to determine ammonia concentrations in the autumn and winter seasons and on weekdays.

## Figures and Tables

**Table 1:** Ambient NH_3_ concentration measurements in the urban atmosphere in different countries/regions.

Location	Period	Methodology	Concentration	Reference
Magurele, Romania	March–August 2021	CO_2_LPAS detector	0.161–134 ppb	Present research
Shanghai, China	April 2014–April 2015	MARGA—online monitor	7.86 ± 5.57 ppb	[48]
New York, USA	April 2016–October 2017	Dual-channel nitric oxide-ozone (NO-O3) chemiluminescence detector system	3.22 ± 2.23 ppb 2.84 ± 1.91 ppb 1.29 ± 1.12 ppb 0.82 ± 0.64 ppb	[48]
Atlanta, USA	July–August 2002	chemical ionization mass spectrometry	0.4–13 ppb	[49]
California, USA	May–June 2010	TES—satellite remote sensing	21 ± 17 ppb	[50]
Huston, USA	12 February 2010–1 March 2010	External cavity quantum cascade laser (EC-QCL)-based sensor employing	2.4 ± 1.2 ppb	[51]
	5 August 2010–25 September 2010	conventional photoacoustic spectroscopy	3.1 ± 2.9 ppb	
Quebec, Canada	2010–2013	Fluorophore membrane filters (PTFE)	0.5–25.01 ppb	[52]
Ontario, Canada	April 2010–March 2011	Willems badge diffusive passive sampler	urban site ~2.86 ppb agricultural site > 4.29 ppb	[53]
Shanghai, China	7 July 2013–30 September 2014	MARGA instrument—online monitor	Industrial: 19.6 ± 8.2 ppb, Rural: 10.4 ± 5.0 ppb), Urban: 5.4 ± 3.3 ppb	[54]
Münster, Germany	30 August 2018–31 October 2018	Chemiluminescence	17 ppb	[55]
North Carolina, USA	18 June–22 August 2002	Tropospheric Emission Spectrometer (TES)	1–6 ppb	[56]
Beijing, China	13 January 2018–13 January 2019	cavity output spectroscopy (OA-ICOS)	urban: 21 ± 14 ppb suburban: 22 ± 15 ppb	[57]

## Data Availability

Not applicable.
